# Clinical features and recurrence of *Corynebacterium kroppenstedtii* infection in patients with mastitis

**DOI:** 10.1186/s12905-022-01859-y

**Published:** 2022-07-06

**Authors:** Weiwei Zeng, Sixian Lao, Wenbin Jia, Xintian Shen, Lijuan Wu, Yan Zhong, Feiling Wang, Guoping Zhong

**Affiliations:** 1 The Second People’s Hospital of Longgang District, Shenzhen, 518112 China; 2 Shenzhen Baoan Women’s and Children’s Hospital, Jinan University, No. 56, Yulv Road, Baoan, Shenzhen, 518102 China; 3 Institute of Clinical Pharmacology, School of Pharmaceutical Sciences, Sun Yat-Sen University, No. 74, Zhongshan 2nd Road, Guangzhou, 510000 China; 4 Guangdong Provincial Key Laboratory of New Drug Design and Evaluation, Guangzhou, 510000 China

**Keywords:** *Corynebacterium kroppenstedtii*, Granulomatous lobular mastitis, Clinical features, Treatment, Antibiotics

## Abstract

**Background:**

Few studies have investigated the differences in clinical features of patients with mastitis following *Corynebacterium kroppenstedtii* infection, and most focused on the bacterial antimicrobial susceptibility, detection methods and therapy.

**Methodology:**

There were 133 patients with mastitis infected by *C. kroppenstedtii* between August 2016 and September 2019. *C. kroppenstedtii* was identified using mass spectrometry. The demographics, clinical diagnosis, laboratory test results of different types of mastitis combined with bacillus infection, and the effects of different treatments in reducing recurrence were compared.

**Results:**

The incidence of pus following *C. kroppenstedtii* infection was higher in patients with non-granulomatous lobular mastitis (NGLM; 56.6%) than in those with granulomatous lobular mastitis (GLM; 33.3%; χ^2^ = 7.072, *p* = 0.008). While C-reactive protein (CRP) was higher in the GLM group (12.50 mg/L) than in the NGLM group (6.05 mg/L; Z =  − 2.187, *p* = 0.029). Treatment with local lavage (triamcinolone acetonide) and antibiotics (cefuroxime) showed a recurrent rate of 25.9% in *C. kroppenstedtii* infection.

**Conclusion:**

Increased pus, large masses, and an elevated CRP level may occur in patients with mastitis infected by *C.kroppenstedtii*. These clinical features may guide the determination of the bacterial infection in patients with mastitis. Combining an antibiotic with a triamcinolone acetonide lavage, preferably cefuroxime, may reduce the recurrence.

**Supplementary Information:**

The online version contains supplementary material available at 10.1186/s12905-022-01859-y.

## Introduction

Mastitis is a benign breast inflammation process with different heterogeneous histopathological findings [1] and the incidence rate has been reported as high as 33% [2]. The term non-puerperal mastitis (NPM) is inflammation not related to pregnancy or lactation. The most prevalent clinical symptoms of NPM are breast tumors and abscesses. Periductal mastitis (PDM) and granulomatous lobular mastitis (GLM) are the two most common pathological types of NPM [3]. In the later stages of NPM, associated fistulas, sinuses, or ulcers can form and are persistent and fail to heal. These conditions have a natural course of 9 to 12 months and frequently reoccur. Moreover, there is no standard treatment available now. GLM is a rare chronic benign breast disease that is characterized by noncaseating granulomas of the breast, often accompanied by abscesses, and its local aggressiveness can cause long-term pain in patients [4]. GLM is more worrisome since it may mimic breast cancer clinically and radiographically, and requires tissue biopsy or surgical excision of the lesion [5]. Core biopsy histopathology or fine-needle aspiration cytology can be used to diagnose GLM and NPM. The GLM individuals with necrotizing granulomas that were accompanied by local infiltration of multinucleated giant cells, epithelioid histiocytes, lymphocytes, and plasma cells that extended to adjacent lobules. Cystic neutrophilic granulomatous mastitis (CNGM) is an uncommon form of granulomatous mastitis linked with Corynebacterium species. CNGM is characterized by suppurative lipogranulomas formed of core lipid vacuoles ringed by neutrophils and an epithelioid histiocyte cuff on the outer surface. Certain lipid vacuoles may host a sparse population of rod-shaped, gram-positive bacteria that are easily seen or disregarded. The mixed inflammatory infiltrate surrounding the lesion is composed of Langhans-type giant cells, lymphocytes, and neutrophils [6]. Parity, current pregnancy and the oral contraceptive pill causing hormonal changes are considered to be contributing factors [7]. *Staphylococcus aureus*, particularly *methicillin-resistant S. aureus* (*MRSA*), is the most common cause of bacterial infection in breastfeeding women and non-lactating mastitis [8, 9]. All the other mastitis in NPM that not belong to GLM was defined as the non-granulomatous lobular mastitis (NGLM). Recently, *C. kroppenstedtii* has been reported to be commonly associated with human breast abscesses and granulomas more and more frequently.

*C. kroppenstedtii* is a lipophilic *Corynebacterium* that was first isolated from sputum specimens in 1998 [10]. *Corynebacterium* comprises nearly 100 species of various gram-positive bacteria. In a study conducted in 2002, the bacterium was detected in samples of 13 patients with breast disease. More than half of them were diagnosed with GLM [11, 12].

Although virulence and pathogenesis of *Corynebacterium* remain unclear in mastitis, the pathogen has been isolated from clinical samples of mammary abscesses in multiple studies [13–16]. *C. kroppenstedtii* infection has become one of the emerging factors for mastitis recurrence in women [17].

Due to the complexity of the diagnosis, patients usually receive antibiotic treatment, frequent biopsies or surgery, including oral prednisone or methotrexate, or surgical resection, before the sensitivities [4, 18]. In addition, mastitis combined with *C. kroppenstedtii* infection often leads to recurrence [17]. Clinical information about the bacterium in Chinese patients is not available. A lack of pathological features makes the diagnosis difficult. These will undoubtedly increase the treatment cost and the patient's discomfort of the patient. It can be seen that it is very necessary to conduct a systematic clinical study on the Chinese patient population to deepen the understanding of the clinical features and treatment of mastitis-associated *C. kroppenstedtii* infection and to provide a theoretical basis for clinical diagnosis.

The current study was undertaken to examine a spectrum of cases with clinical diagnosis of mastitis infected with *C. kroppenstedtii* who have been admitted to a specialist hospital in Shenzhen. The purpose of this study was to identify the clinical features of mastitis patients with *C. kroppenstedtii* infection, and compare the efficacy of different treatments on mastitis patients with *C. kroppenstedtii* infection, including local lavage (triamcinolone acetonide) combined with cefuroxime.

## Methods

### Patients and diagnosis

The current study was undertaken on cases with a clinical diagnosis of mastitis infected with *C. kroppenstedtii* who have been admitted to a specialist hospital in Shenzhen. In this retrospective record-based study, 133 patients with microbiological evidence of *C. kroppenstedtii* infection admitted at Baoan Women’s and Children’s Hospital in Shenzhen, China, from August 2016 to September 2020. Only one of the included patients had lactating mastitis. Our study mainly focused on NPM, especially granulomatous mastitis and non-granulomatous mastitis. All procedures performed in this study involving human participants were in accordance with the Declaration of Helsinki (as revised in 2013). This retrospective study was approved by the Institutional Review Board of Baoan Women’s and Children’s Hospital, Shenzhen, China with IRB No LLSCHY2021-1-08 and individual consent was waived.

Clinical information was obtained from the hospital’s electronic database. Patients' demographics (age, weight, clinical diagnosis, and history of pregnancy and lactation), laboratory test results (white blood cell [WBC] count, neutrophil granulocyte [NEUT], lymphocyte [LYMPH], and C-reactive protein [CRP]), clinical treatment, and recurrence during or after treatment were systematically described and analyzed. If the patient is re-admitted to the hospital for treatment for the same reason, it is defined as a recurrence. All patients were followed up for four years and called for asking about whether recovered.

The patients whose complete body mass index (BMI) information was available were divided into low-weight (BMI < 18.5; 1 [1.6%]), normal-weight (BMI = 18.5–24; 40 [59.7%]), and obese (BMI > 24; 21 [38.7%]) groups according to the Guidelines for the Prevention and Control of Overweight and Obesity in Chinese Adults [12]. According to the doctor’s experience and visual judgment, 2 × 2 cm^2^ was defined as a large mass.

The diagnosis of GLM is made by core biopsy histopathology or fine-needle aspiration cytology. The histopathological features of patients with necrotizing granulomas, accompanied by local infiltration of multinucleated giant cells, epithelioid histiocytes, lymphocytes and plasma cells, and extending to adjacent lobules, were included in the GLM group. Any other patients who have been diagnosed with nonlubolocentric granuloma mastitis are included in the NGLM group.

## Intervention

Before the results of drug sensitivity came out, patients received conventional steroid and antibiotic treatment based on experience and symptoms, mainly including triamcinolone acetonide acetate and cefuroxime. For patients with scattered pus, doctors usually arrange triamcinolone acetonide lavage in the early stages of treatment. If the symptoms are not relieved, the choice of antibiotics is based on the results of drug susceptibility tests with pus. The patient received steroids (40 mg of triamcinolone acetonide acetate injection each time *local lavage), and all selected antibiotic, according to drug susceptibility results, were included in analysis including amoxicillin and clavulanate potassium tablets 457 mg q12h; doxycycline tablets 0.1 g bid; gentamicin sulfate injection 160,000 IU for lavage; cefuroxime axetil tablets 0.25 g bid; cefixime capsules 0.1 g bid; levofloxacin hydrochloride capsules 0.2 g bid; penicillin sodium for injection 800,000 IU; cefuroxime sodium for injection 1.5 g q8h; ceftriaxone sodium for injection 1 g qd; levofloxacin injection 0.2 g bid.

### Microbiological assays

**Fig. 1:**
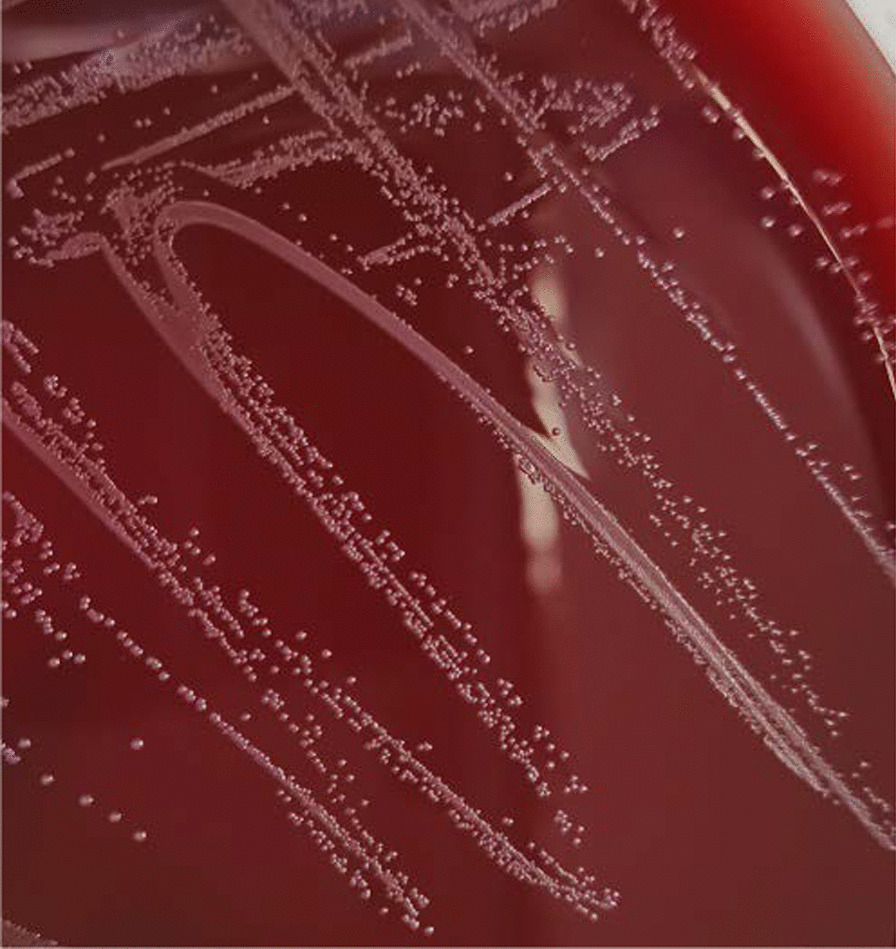
*Corynebacterium kroppenstedtii* after isolated and subculture on sheep blood agar at 48 h

The breast pus samples of patients were collected and cultured on 5% sheep’s blood agar plate (BAP), chocolate agar plate and McConkey agar plate (Guangzhou Detgerm Microbiological Science Ltd, China) and incubated at 35 °C and 5% CO_2_ for 5 days. *C. kroppenstedtii* isolates showed grayish, translucent, slightly dry, and less than 0.5 mm in diameter on BAP with enrichment approximately 48–72 h (Fig. 1) and were identified by matrix-assisted laser desorption/ionization time-of-flight mass spectrometry (MALDI-TOF MS) using the Bruker Biotyper Compass library 10.0 [15, 19, 20]. The E-test (Wenzhou Kangtai Biotechnology, China) using a BAP incubated at 5% CO_2_ and 35 °C for 24–48 h, which demonstrated susceptibility to vancomycin, linezolid, rifampin, meropenem, gentamicin, ciprofloxacin, ceftriaxone, tetracycline, clindamycin, penicillin, erythromycin and trimethoprim-sulfamethoxazole. The Clinical and Laboratory Standards Institute (CLSI) M45-A3 guideline for Corynebacterium species was used for conducting the sensitivity interpretation.

### Statistical analysis

Data are presented as median (Q1-Q3) for continuous variables and as number or percentage for categorical variables. Laboratory results between the groups were compared using the Mann–Whitney U test and multivariate logistic regression analysis. The chi-square test was used to compare differences between the groups. A *p* value of < 0.05 was considered statistically significant. All statistical analyses were performed using the SPSS statistical software package version 25.0 (IBM, Armonk, NY).

## Results

Between August 2016 and September 2020, a total of 133 patients, aged 11 to 56 years old, were diagnosed with *C. kroppenstedtii*-infected mastitis, with one case diagnosed as lactation mastitis. Seventy six (57.1%) and fifty seven (42.9%) patients were diagnosed as having NGLM and GLM, respectively. The details of clinical diagnosis, treatment, laboratory test results, and recurrence were presented in Table 1.

**Table 1:** Clinical characteristics of patients infected by *Corynebacterium kroppenstedtii*

Characteristic	N = 133	NGLM (n = 75)	GLM (n = 57)	*P*
	(All were female patients)	(Exclude one lactation mastitis patients)		
Age in years, median (range)	32 (11–56)	31 (29–34)	32 (29–35)	0.43
Weight in kg, median (range)	59.8 (43.0–88.0); n = 98	58.0 (52.5–64.5); n = 41	57.8 (53.0–66.0); n = 56	0.88
BMI in kg/m^2^, median (range)	23.0 (16.8–32.4);n = 62	22.1 (20.3–25.0); n = 20	22.5 (20.9–24.9); n = 41	0.12
Low weight, < 18.5	1 (1.6%)	0 (0.0%)	1 (2.4%)	
Normal weight, 18.5–24	40 (59.7%)	10 (50.0%)	30 (73.2%)	
Obesity, ≥ 24	21 (38.7%)	10 (50.0%)	10 (24.4%)	
Clinical presentation				
Nonlobular granulomatous mastitis	76 (57.1%)			
Lobular granulomatous mastitis	57 (42.9%)			
Laterality	n = 133			0.74
Left	63 (47.4%)	33 (44.0%)	29 (50.9%)	
Right	60 (45.1%)	36 (48.0%)	24 (42.1%)	
Bilateral	10 (7.5%)	6 (8.0%)	4 (7%)	
Symptoms				
Pain	120	68 (89.5%)	52 (91.2)	0.74
Pus	52	43 (56.0%)	19 (33.3%)	0.01^*^
Mass	131	74 (97.4%)	57 (100.0%)	0.61
Two or more of the above symptoms	127	74 (94.9%)	55 (96.5%)	0.65
*Laboratory test result, median (range)*				
WBC, × 10^9^/L	9.3 (4.1–18.7); n = 107	8.95 (6.81–11.05)	8.95 (7.45–11.05)	0.74
NEUT, × 10^9^/L	7.3 (2.0–78.4); n = 106	5.75 (4.39–8.16)	6.40 (5.11–8.43)	0.27
LYMPH, × 10^9^/L	2.03 (0.58–16.30); n = 106	1.85 (1.43–2.29)	1.69 (1.38–2.17)	0.45
CRP, mg/L	15.9 (0.5–109.4); n = 66	6.05 (4.10–33.40)	12.50 (1.50–10.30)	0.03^*^
Medical History				
Hypertension	1	0	1	
Mammary gland tumor	3	1	2	
Depression	1	0	1	
Childbirth history	n = 116			0.68
0	4	2 (3.3%)	2 (3.6%)	
1	71	34 (56.7%)	37 (67.3%)	
2	36	21 (35.0%)	14 (25.5%)	
3	5	3 (5.0%)	2 (3.6%)	
History of lactation in months, median (range) 10.5 (0–48); n = 110				0.15
< 12	70	33 (61.1%)	37 (67.3%)	
12–24	32	19 (35.2%)	12 (21.8%)	
> 24	8	2 (3.7%)	6 (10.9%)	
Clinical Treatment with Medications	n = 114			< 0.01*
Steroid	22	9 (16.7%)	13 (22.0%)	
Antibiotic	56	40 (74.1%)	15 (25.4%)	
Steroid and antibiotic	36	5 (9.3%)	31 (52.3%)	
Invasive Clinical Treatment	n = 64			0.01^*^
Punctures	29	7 (38.9%)	22 (48.9%)	
Drainage	13	8 (44.4%)	5 (11.1%)	
Punctures and drainage	22	3 (16.7%)	18 (40.0%)	
Recurrence	n = 105			0.90
0	59	28 (58.3%)	30 (53.6%)	
1	32	13 (27.1%)	19 (33.9%)	
2	10	5 (10.4%)	5 (8.9%)	
≥ 3	4	2 (4.2%)	2 (3.6%)	

*WBC* white cell count, *NEUT* neutrophil granulocyte, *LYMPH* lymphocyte, *CRP* C-reactive protein, *GLM* granulomatous lobular mastitis, *NGLM* non-granulomatous lobular mastitis

*p < 0.01 which means there is significant difference between groups

### Demographics

All the patients were female and the median age was 32 years old. Most patients (n = 28 [70.0%]) were in the normal-weight patient group and diagnosed as having GLM, and the incidence of GLM was higher than in the NGLM group in the normal-weight patients. The patients with a history of childbirth showed a higher prevalence of GLM (52.1%). However, the incidence of GLM did not demonstrate a statistically significant correlation with age (χ^2^ = 0.035, *p* = 0.852), BMI (χ^2^ = 1.145, *p* = 0.285), childbirth history (χ^2^ = 1.810, *p* = 0.613), or lactation history (χ^2^ = 4.343, *p* = 0.114), but the incidence of GLM was higher (n = 6 [75%]) in patients with a lactation history of > 24 months. Clinical symptoms mainly included pain, pus, and masses.

### Treatment

A total of 141 individuals were given medicine as well as partial physical cooling using wet and cold towels on the breast. In 56 individuals, antibiotics were the most usually prescribed drug. Breast piercing was performed on a total of 36 individuals, including all those with a history of steroid usage.

After excluding the one patient with lactation mastitis, all cases were classified into different treatment groups, including steroids-only, antibiotics-only, and antibiotic-steroid groups. The recurrence rate was found to be higher in the steroids-only group (48.5%) than in the antibiotic-steroid (37.8%) and antibiotics-only (25.0%) groups, but no significant was found (**χ**^**2**^ = 4.83, p = 0.09) (Table 2).

**Table 2:** The recurrence of different treatment groups

Treatment group	Without recurrence (%)	Recurrence (%)	χ^2^	*p* value
Steroid	17 (51.5)	16 (48.5)	4.83	0.09
Antibiotic	36 (75.0)	12 (25.0)		
Steroid and antibiotic	28 (62.2)	17 (37.8)		

**Fig. 2:**
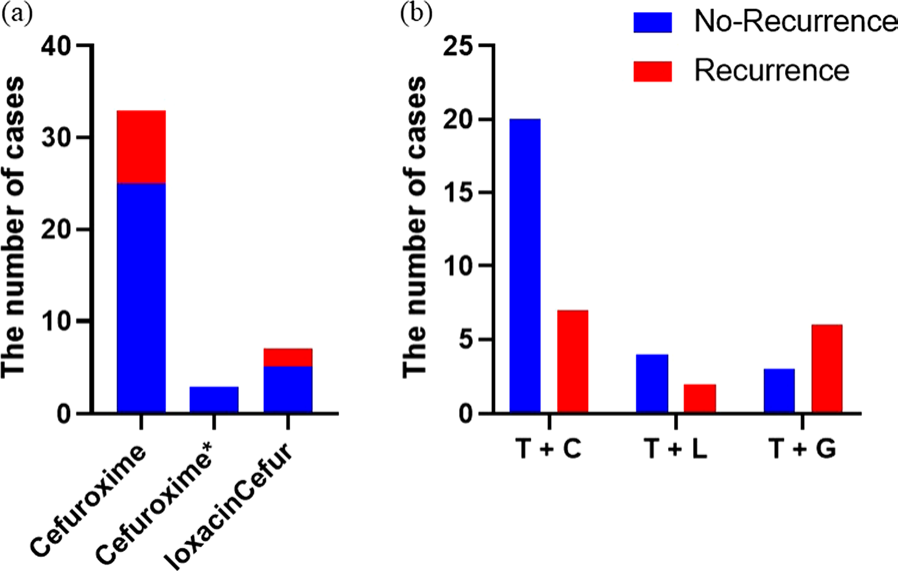
The recurrence of mastitis treated with different antibiotics **a** or steroid and antibiotics **b**. Cefuroxime*: Cefuroxime + doxycycline or ceftriaxone or ciprofloxacin; T + C: triamcinolone acetonide + gentamicin; T + L: triamcinolone acetonide + levofloxacin; T + G: triamcinolone acetonide + cefuroxime

Cefuroxime was the most commonly prescribed antibiotic in the antibiotic-only group. There were 24 (75.0%) cases that recovered without recurrence and 8 instances that experienced recurrence among the thirty-two patients that treated only cefuroxime. Three cases that were treated with antibiotics such as doxycycline, ceftriaxone, and ciprofloxacin did not recur. Five of the seven patients treated with levofloxacin did not have a recurrence (Fig. 2). One of the two patients treated with amoxicillin-clavulanic acid had a recurrence. Patients who took Doxycycline tablets later relapsed. Only vancomycin was used in one case, and the patient recovered entirely.

The only steroid used in the antibiotic-steroid group was triamcinolone acetonide, and the antibiotics were cefuroxime (27 patients), levofloxacin (6 patients), or gentamicin (8 patients). 27 cases were treated with a combination of triamcinolone acetonide and cefuroxime, with a recurrence rate of 25.9%, which was lower than those who received a combination of triamcinolone acetonide and levofloxacin (33.3%) or triamcinolone acetonide and gentamicin (66.7%; Fig. 2). One patient who had triamcinolone acetonide and doxycycline experienced recurrence. Even after treatment triamcinolone acetonide and amoxicillin-clavulanic acid, mastitis recurred in one patient.

### GLM versus NGLM

After omitting the one patient with lactation mastitis, NGLM was found in 75 (56.8%) of the patients, while GLM was found in 57 (43.2%) of the patients. GLM was diagnosed in a total of 57 cases. 45 individuals had visible lumps, and 21 patients had pus, according to clinical diagnosis. Thirty-one patients with GLM did not undergo treatment again because of recurrence, and twenty-six individuals had recurrence.

Ninety-four patients had obvious masses in the clinical examination. Overall, 39 (51.3%) patients with NGLM had large masses (> 2 × 2 cm^2^) and 36 (63.2%) patients with GLM had large masses. Although no statistical difference was observed between these groups (*p* = 0.173), patients with GLM were more likely to develop a large mass than patients with NGLM, 63.2% and 51.3% respectively. There are differences in the selection of drugs (*p* < 0.001) and surgical interventions (*p* = 0.010) by doctors. Among NGLM patients, the most patients receive antibiotic therapy only (74.1%), and in GLM patients, more than half receive antibiotics and steroids (52.3%). Punctures (38.9%) or drainage (44.4%) were the main interventions for NGLM patients. Among GLM patients, the most commonly used intervention is punctures (48.9%).

A significant higher incidence of pus was found in patients with NGLM (*p* = 0.008, 56.6% vs 33.3%). However, the incidence of other symptoms was not found to differ significantly between the two groups (pain, *p* = 0.736; mass, *p* = 0.607; two or more symptoms, *p* = 0.978; Table 1).

The laboratory variables between the two groups, were found to have no difference, such as WBC, (8.95 [6.81–11.05] = 8.95 [7.45–11.05];* p* = 0.736); NEUT, (5.75 [4.39–8.16] < 6.40 [5.11–8.43];* p* = 0.268); LYMPH, (1.85 [1.43–2.29] > 1.69 [1.38–2.17];* p* = 0.450). While CRP showed a significant higher the GLM group (12.50 [1.50–10.30]) compared to the NGLM group (*p* = 0.029, 6.05 [4.10–33.40]; see Table 1). Multivariate analysis did not show significant differences between the two groups (Table 3).

**Table 3:** The multivariable analysis of laboratory test result between groups

Variables	NGLM	GLM	Multivariable			
	n	n	OR	95% CI		*P*
				Lower	Upper	
White cell count	57	49	0.75	0.34	1.69	0.49
Neutrophil granulocyte	57	48	1.37	0.55	3.44	0.50
Lymphocyte	66	39	0.85	0.50	1.44	0.54
C-reactive protein	38	27	1.00	1.00	1.03	0.81

*GLM* granulomatous lobular mastitis, *NGLM* non-granulomatous lobular mastitis,CI

### Recurrence of GLM

There were 30 patients without recurrence and 27 patients whose recurrence was more than once in the patients diagnosed as GLM. No significant difference was found in the incidence of pus between the two groups (*p* = 0.816). Moreover, there were no significant differences in WBC (*p* = 0.132), NEUT (*p* = 0.140), CRP (*p* = 0.339) or LYMPH (*p* = 0.714) between these two groups (data didn’t show).

Compare to the recurrence after receiving one type of drug (steroid or antibiotic) and receiving two drugs at the same time, there was no significant difference in the recurrence rate of different treatment options between the two groups (*p* = 0.630) (data didn’t show) (Table 2).

### Antibiotic sensitivity test

*C. kroppenstedtii* E-test MICs were read at 48 h. Due to limited laboratory testing conditions, only 56 patients had the antibiotic sensitivity in all the patients with microbiological evidence of *C. kroppenstedtii*. The antibacterial susceptibility results were shown in Table 4.

**Table 4:** Antimicrobial intermediary and antimicrobial susceptibilities of 56 patients

Drug	Susceptible %	Intermediate %	Resistant %	MIC_50_	MIC_90_	Breakpoint
Vancomycin	100 (49/49)	0.00 (49/49)	0.00 (49/49)	0.5	0.5	S ≤ 2
Linezolid	100 (38/38)	0.00 (38/38)	0.00 (38/38)	0.25	0.5	S ≤ 2
Rifampin	100 (27/27)	0.00 (27/27)	0.00 (27/27)	0.008	0.008	S ≤ 1, R ≥ 4
Meropenem	87.50 (42/48)	4.17 (2/48)	8.33 (4/48)	0.064	0.5	S ≤ 0.25, R ≥ 1
Gentamicin	94.12 (32/34)	0.00 (0/34)	5.88 (2/34)	0.125	0.5	S ≤ 4, R ≥ 16
Trimethoprim -sulfamethoxazole	77.36 (41/53)	0.00 (0/53)	22.64 (12/53)	0.25	32	S ≤ 2/38, R ≥ 4/76
Ciprofloxacin	69.09 (38/55)	0.00 (0/55)	30.91 (17/55)	0.25	32	S ≤ 1, R ≥ 4
Ceftriaxone	50.00 (28/56)	14.29 (8/56)	35.71 (20/56)	1	8	S ≤ 1, R ≥ 4
Tetracycline	41.46 (17/41)	48.78 (20/41)	9.76 (4/41)	8	8	S ≤ 4, R ≥ 16
Clindamycin	23.26 (10/43)	0.00 (0/43)	76.74 (33/43)	256	256	S ≤ 0.5, R ≥ 4
Erythromycin	19.64 (11/56)	1.79 (1/56)	78.57 (44/56)	16	256	S ≤ 0.5, R ≥ 2
Penicillin	0.00 (0/56)	89.29 (50/56)	10.71 (6/56)	0.5	2	S ≤ 0.12, R ≥ 4

MIC: minimum inhibitory concentration

In general, *C. kroppenstedtii* demonstrated a high sensitivity to gentamicin, meropenem, cotrimoxazole, ciprofloxacin, vancomycin, gentamicin, and rifampicin. Typically, these antibiotics are preferred in clinical practice.

## Discussion

The present study is the first to review the largest cohort of Chinese patients with *C. kroppenstedtii* infection [16, 18, 21–23]. This study investigated the clinical features of *C. kroppenstedtii* infection in Chinese patients and explored the efficacy of local lavage (triamcinolone acetonide) and antibiotics (cefuroxime).

Due to the special newness of breast tissue, the normal flora of the breast contains multiple microorganisms. *C. kroppenstedtii* is a rare lipophilic *Corynebacterium* but is commonly found in fat-rich breasts because of its dependence on lipids for growth and reproduction [24]. Clinical data has confirmed the relationship between corynebacterial infection and granulomatous mastitis [12]. As the condition becomes more complicated after mastitis infection with *C. kroppenstedtii*, it is often accompanied by relapse and difficult diagnosis [17, 18]. Clinical symptoms and efficacy of various therapies in mastitis with *C. kroppenstedtii* are limited, especially in Chinese patients. Lack of adequate research on these aspects typically can leads to a delay in diagnosis and optimal treatment selection, increasing the psychological pressure of patients and the cost of treatment.

In our research, the most common clinical symptoms in patients with *C. kroppenstedtii* infection were pain, pus, and masses. In addition, our research also showed that GLM and NGLM infected by *C. kroppenstedtii* show different clinical features. Further classification and analysis revealed that patients with GLM and NGLM had a high incidence of pain and mass. However, only the incidence of pus was found to differ significantly between the two groups. The results also provide a theoretical basis for determining whether a patient is infected with *C. kroppenstedtii*, when NGLM patients have symptoms of pus, the doctor should consider the risk of the patient being infected with *C. kroppenstedtii* infection.

According to laboratory results of relevant inflammatory factors, the results indicate that the *C. kroppenstedtii* infection affects the physiological indicators in patients with different types of breast mastitis. The median value of CRP was found to differ significantly between the GLM and NGLM groups in this study. Elevated CRP levels in GLM patients may suggest *C. kroppenstedtii* infection, leading to timely diagnosis and optimization of the therapeutic regimen.

The above-mentioned differences in clinical characteristics and laboratory indicators between GLM and NGLM have not been widely reported, indicating that infectious bacilli do have unexpected effects on different types of mastitis, although from our research, the relationship between bacillus infection and the cause of GLM cannot be drawn, but it was proved that bacillus infection can complicate the original mastitis.

Currently, the 16S-rRNA-sequencing approach is being widely used to identify *C. kroppenstedtii* [25]. The approach is particularly for the identification of rare and difficult bacterial species from clinical specimens and has advantages of rapid detection and cost-effectiveness. Mass spectrometry, used to detect the bacterium in this study, is also an economical, rapid, and accurate method with clinically confirmed reliability and accuracy.

Lipophilic antibiotics with a high volume of distribution, such as doxycycline, clarithromycin, and rifampicin, may reach adequate therapeutic concentrations within the lipogranulomas of the lipid-rich breast. An antibiotic-steroid regimen may be beneficial in inhibiting the development of granulomatous disease, although evidence supporting the efficacy of this combination regimen is insufficient. Further studies are required to support the use of this combination regimen.

In contrast to the previous report, most of the patients in this study treated with surgical drainage with antibiotic-steroids brought resolution of symptoms. Before the results of drug sensitivity are available, clinicians often recommend broad-spectrum antibacterial drugs (such as cefuroxime) based on experience and habits. According to the drugs received by the included patients, compared with NGLM patients, antibiotics and steroids were most commonly received in GLM patients infected with bacilli, which also suggested that infection may be one of the factors that complicate the disease. Limited by the small number of samples included, this study failed to conclude on an optimal therapy for breast mastitis with corynebacterial infection. However, data suggests that a combination regimen of a steroid and an antibiotic offers obvious advantages in term of improved therapeutic efficacy and reduced disease recurrence. Cephalosporins, especially cefuroxime, seem to be the most potent antibiotic, whereas doxycycline and amoxicillin-clavulanic acid have limited therapeutic effects in patients with *C. kroppenstedtii* infection. In our study, recurrence occurs in patients treated with doxycycline alone or in combination with steroids. Moreover, disease recurrence was noted in two patients who received amoxicillin-clavulanic acid (a total of three patients). In the case we observed, recurrence occurred in one case treated with amoxicillin-clavulanic acid, and doxycycline tablets were used to treat one case, in whom the disease relapsed later. After receiving triamcinolone acetonide and amoxicillin-clavulanic acid treatment, one case of mastitis recurred. This is consistent with reports that after receiving amoxicillin-clavulanic acid and doxycycline treatment, the symptom relief effect is poor and even recurrence occurs, indicating that the recommended level of the drug for mastitis complicated with Bacillus infection is low[10]. In summary, the combination of a steroid (triamcinolone acetonide) and an antibiotic (cefuroxime) may be considered the first choice for treating *C. kroppenstedtii* infections in our data.

## Limitation

This study has certain limitations. Although data of 133 patients were included, the clinical information for a few patients was missing and a few patients were noncompliant with timely follow-ups. There may be some different radiologic features in these patients, but because of different clinical data collection system, we cannot obtain the original radiological examination report of each patient. In the analysis of some indicators, data of certain patients were excluded, which could have resulted partly in a bias in the final results and thus necessitates supplementation and corroboration of results with subsequent clinical information. In addition, this study did not include patients using different steroids and different methods of administration, which makes it impossible to compare their effects.

## Conclusion

Our study provided data for further research into this infection in Chinese patients, making up for the lack of clinical information on *C. kroppenstedtii* infection in the Chinese population. The study results suggested that patients with NGLM are more likely to produce pus than GLM, and the level of CRP was higher in patients with GLM than NGLM, after infection with *C. kroppenstedtii*. According to comparing different therapies, it is worth noting that a combination of cefuroxime and triamcinolone acetonide lavage, has better therapeutic effects and can effectively reduce the recurrence of mastitis.

## Supplementary Information

**Additional file 1**. Corynebacterium kroppenstedtii database and Please insert into the section of Data availability.

## Data Availability

The datasets generated and/or analysed during the current study are available in Additional file 1.
